# Determination of the relationship between doxorubicin resistance and Wnt signaling pathway in HeLa and K562 cell lines

**DOI:** 10.17179/excli2018-1129

**Published:** 2018-05-02

**Authors:** Pelin Mutlu, Serap Yalçin Azarkan, Negar Taghavi Pourianazar, Meral Yücel, Ufuk Gündüz

**Affiliations:** 1 Central Laboratory Molecular Biology and Biotechnology R&D, Middle East Technical University, Ankara, Turkey; 2 Department of Molecular Biology and Genetics, Ahi Evran University, Kirsehir, Turkey; 3 Department of Biological Sciences, Middle East Technical University, Ankara, Turkey

**Keywords:** Wnt signaling pathway, drug resistance, cervical cancer, chronic myelogenous leukemia, gene expression

## Abstract

Activation of the Wnt signaling in some types of cancer and its relation with chemotherapy resistance is a very interesting issue that has been emphasized in recent years. Although, it is known that increase in the activity of β-catenin is important in blast transformation and drug resistance, the underlying mechanisms are still unclear. In this study, changes in the expression levels of 186 genes that are thought to be important in drug resistance and Wnt signaling pathways were determined by using qPCR method in doxorubicin-sensitive and -resistant HeLa and K562 cell lines. It has been observed that the genes involved in the Wnt signaling pathways are involved in more changes in HeLa/Dox cells (36 genes) than in the K562/Dox cells (17 genes). Genes important for the development of cancer resistance have been found to be significantly different in expression levels of 18 genes in HeLa/Dox cells and 20 genes in K562/Dox cells. In both cell lines, the expression of ABCB1 gene was significantly increased to 160 and 103 fold, respectively. However, despite the resistance to same drug in HeLa and K562 cell lines, it appears that the expression levels of different oncogenes and genes involved in Wnt signaling pathways have been altered. It has been found that although resistance develops to the same drug in both cell lines, the expression levels of different genes have changed. If functional analysis of these genes is performed on patient population groups, these molecules may become candidates for novel therapeutic target molecules.

## Introduction

Wnt signaling pathway is involved in both embryonic and adult stages in an organism (Nusse and Varmus, 1992[31]; Nusse, 2005[28]). Wnt signaling pathways play important roles in the adhesion of self-renewing cells in adults, the control of transcription of target genes and the cell polarity, proliferation, differentiation and cell migration in cells in the embriyonic period (Willert and Nusse, 1998[37]; Miller, 2002[25]; Mikels and Nusse, 2006[24]). In particular, the relationship of the Wnt/β-catenin signaling pathway to cancer has been a very interesting issue that has been addressed in recent years. The role of Wnt proteins in cancer was first demonstrated in the breast cancer mouse model (Nusse and Varmus, 1982[30]; Nusse et al., 1984[29]). ß-catenin is found in the cytoplasm and is transported to the nucleus by Wnt signal activation (Ilyas, 2005[13]). Thus, it activates transcription of a large number of genes that regulate cell proliferation and development. Many of these genes are known as oncogenes and are powerful targets in the treatment of cancer. Changes in the gene expression levels of molecules involved in this signal pathway can lead to the emergence of many cancer types (Lustig and Behrens, 2003[21]). 

Chemotherapy is widely used in the treatment of cancer. However, the failure of patients to respond to treatment during chemotherapy or the recurrence of cancer after treatment is a condition that seriously affects the success of chemotherapy (Krishan et al., 1997[17]). This is referred to as multidrug resistance (Ueda et al., 1987[36]). Several mechanisms responsible for drug resistance have been identified. These include increase in gene expression levels of members of the ATP-binding cassette (ABC) transporter family members, deoxycytidine kinase-like enzyme modifications, inhibition of apoptosis and changes in target enzyme of the cytotoxic drugs (Marie, 2001[22]). 

The multidrug resistance 1 gene (MDR1) encodes an ABC transporter called P-glycoprotein (P-gp), which is the direct target of the Wnt/β-catenin signaling pathway. Overexpression of P-gp actively pumps out cytotoxic drugs from the cell and by reducing drug concentration in the cell cause drug resistance. The Frizzled 1 protein (FZD1), an important member of the Wnt/β-catenin pathway, and P-gp have been shown to increase in the adriamycin resistant MCF-7 breast cancer cell line. It has been shown that when the FZD1 gene expression is silenced via siRNA, cytoplasmic and nuclear β-catenin levels fall, as well as the level of P-gp decreases and sensitivity to chemotherapeutic agents is regained (Zhang et al., 2012[40]). 

Activation of Wnt signaling is known to play a role in the pathogenesis of some solid tumors and leukemia types (Lu et al., 2009[20]; Prosperi and Goss, 2010[33]; Chen et al., 2010[5]; Ge and Wang, 2010[8]). Flahaut et al. and Bourguignon et al. have shown that the Wnt/β-catenin pathway is important in ABCB1 regulation in colorectal cancer, neuroblastoma, and breast cancer (Flahaut et al., 2009[7]; Bourguignon et al., 2009[2]). It is now known that the increase in β-catenin activity is important for blast transformation and drug resistance, but the underlying mechanisms are still largely unknown.

In this study, 186 genes that are related to doxorubicin resistance and Wnt signaling pathway were selected and gene expression profiles were studied in sensitive and doxorubicin resistant HeLa and K562 cell lines. The selection of model cell lines for two different types of cancer is to demonstrate possible similar and/or different effects of the same drug in different types of cancer. The data obtained from this study aimed to reveal new diagnostic, prognostic and therapeutic candidate molecules that are important in the Wnt signaling pathway for doxorubicin resistance.

## Materials and Methods

### Cell culture and cytotoxicity analysis

Doxorubicin resistant variants of original HeLa and K562 cell lines were developed in our laboratory by applying the drug in dose increments. The cells were grown in 25 cm^2^ culture dishes in RPMI 1640 medium supplemented with 10 % fetal bovine serum, incubated at 37 °C in a humidified air atmosphere with 5 % carbon dioxide incubator. Doxorubicin resistant variants were determined by XTT cytotoxicity analyzes by using Cell Proliferation Assay Kit (Biological Industries, Isreal). IC_50_ values of both original and drug resistant cells were determined. 

### RNA isolation and cDNA synthesis

RNA isolation was performed from HeLa and K562 cell lines, which are sensitive and resistant to doxorubicin using TRI Reagent (Sigma, St Louis, MO, USA) according to the manufacturer's instructions. RNA samples were assessed qualitatively on an Agilent 2100 Bioanalyzer. RNA samples with sufficient concentration (at least 200 ng/µl) and free of genomic DNA and protein contamination were used for cDNA synthesis. cDNAs were synthesized from total RNA by using random primers of Transcriptor High Fidelity cDNA Synthesis Kit (Roche Life Sciences) according to manufacturer's instructions.

### Quantitative RT-PCR

PCR-arrays (RealTime ready Custom qPCR Assays, Roche Life Sciences) were performed on 96-well plates using the quantitative RT-PCR method by using Roche Light Cycler 480 instrument. It has been attempted to determine the expression levels of the genes which have a role in Wnt signaling pathways and genes which are thought to be important in resistance development. Genes on the "Wnt Signal Transduction PCR Array" and "Drug Resistance PCR Array" are given in Tables 1(Tab. 1) and 2(Tab. 2), respectively.

### Statistical analysis

RT-PCR analyzes were performed in dublicates using a total of 16 PCR arrays from both the "Wnt Signal Transduction PCR array" and the "Drug Resistance PCR array" from all original and doxorubicin resistant cell lines. Relative amount changes were determined by calculating the Ct values (2-ΔΔCt) obtained for each gene. According to this method; from a Ct value relative to a control gene (beta-actin) in a gene-resistant drug line; the value of ΔΔCt [ΔΔCt = ΔCt resistant-ΔCt original] was obtained by subtracting the Ct value relative to the control gene in the same cell line. The fold change in the amount of gene was obtained by substituting ΔΔCt in the 2-ΔΔCt. The results obtained with the two replicates were evaluated with the SPSS 16.0 program and the differences less than the p-value of 0.05 and those with more or less 2 folds changes in gene expression levels were considered as "statistically significant".

## Results

### XTT cytotoxicity analysis

**Figure 1:**
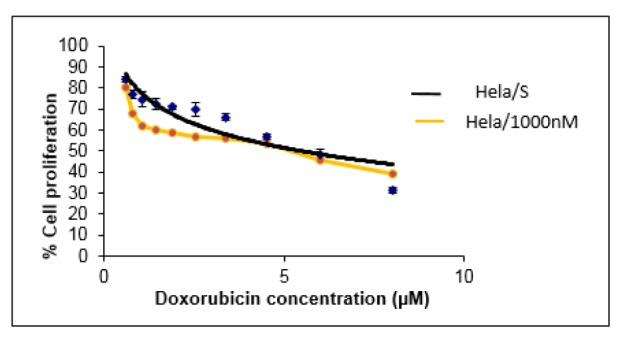
Antiproliferative effect of doxorubicin on original and doxorubicin resistant HeLa cell lines

**Figure 2:**
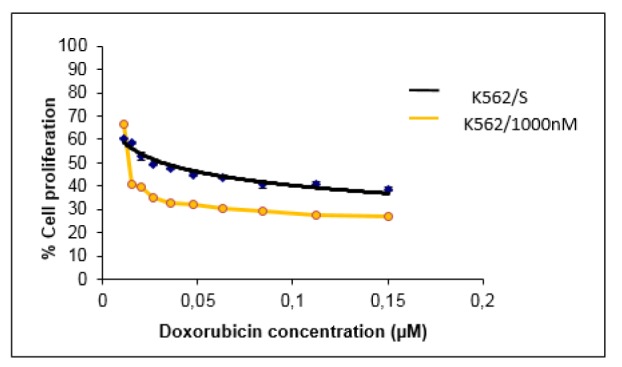
Antiproliferative effect of doxorubicin on original and doxorubicin resistant K562 cell lines

The degree of doxorubicin resistance in HeLa and K562 cells was determined by measuring the IC_50_ values at 72 h by XTT assay. In the original HeLa cell line, doxorubicin killed 50 % of the cells at a dose of 2,664 μM (IC50 = 2,664 μM) whereas in doxorubicin-resistant HeLa cells this value was found to be 5,470 μM (IC_50_ = 5,470 μM) (Figure 1(Fig. 1)). These results show that the doxorubicin-resistant HeLa cell line is about 2 times more resistant than the original HeLa line. On the other hand, in the original K562 cell line, the dose of doxorubicin killing 50 % of the cells was 0.031 μM (IC_50_ = 0.031 μM), while in doxorubicin-resistant K562 cells this value was found to be 0.996 μM (IC_50_ = 0.996 μM). These results show that doxorubicin-resistant K562 cell line is approximately 32 times more resistant than the original K562 line (Figure 2(Fig. 2)).

### Wnt signaling pathway PCR array

Table 1(Tab. 1) summarizes the alterations in expression levels of the genes in Wnt signaling pathway in doxorubicin resistant HeLa and K562 cell lines. According to these results; among 93 genes the expression levels of 36 genes in HeLa/Dox and 17 genes in K562/ Dox were observed to change with respect to original cell lines. Doxorubicin-resistant K562 cells did not show a significant change in expression of CTNNB1 gene encoding beta-catenin; as well as an increase of about 8-folds in the gene encoding FZD1 from Wnt receptors and a 15-folds decrease in the DAB2 gene and a 3-folds decrease in the SOCS3 gene were determined, which are known as tumor suppressor genes. Furthermore, it has been determined that there is a significant decrease in expression of the AXIN2 gene found in the beta-catenin degradation complex and in the expression of the CXXC4 gene which codes for a Wnt antagonist. When we examined HeLa/Dox cells, it was generally found that there was a decrease in the expression of Wnt receptor genes. 

### Drug resistance PCR array

Table 2(Tab. 2) summarizes the alterations in expression levels of genes that are related to drug resistance in doxorubicin resistant HeLa and K562 cell lines. The expression of the ABCB1 gene encoding MDR1 protein in both cell lines was significantly increased to 160 and 103 folds, respectively. On the other hand, the decrease in expression of the ABCC1 gene encoding the MRP1 gene in both cell lines suggests that the doxorubicin resistance can be due to the increase in the MDR1 gene rather than MRP1. According to the results, it is seen that the different oncogene expressions have changed in doxorubicin resistant HeLa and K562 cell lines in spite of the resistance gained to the same drug. An increase in Fos gene expression was observed in HeLa/Dox, whereas Myc gene overexpression was detected in K562/Dox cell line. In addition, genes coding for proteins involved in cell cycle, apoptosis, and drug metabolism (CDKN1A, CCND1, BAX, FAS), growth factors and its receptors (FGF2, IGF1R, IGF2R, MET), different transcription factors (HIF1A, NFKB1, NFKB2, RANK), CYP2E1, CYP3A5) were found to be significantly different between doxorubicin-resistant HeLa and K562 cell lines.

**Figure 3:**
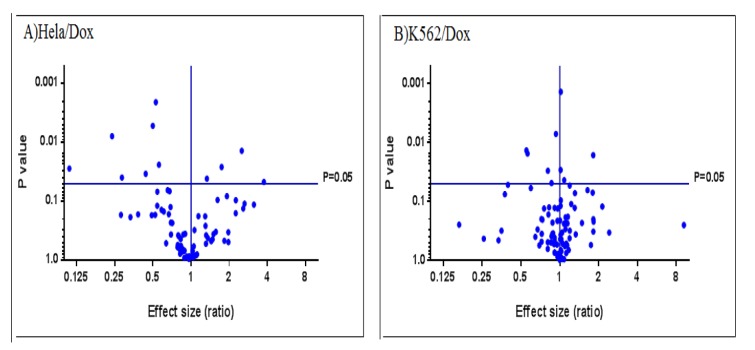
Volcano plots displaying differential expressed genes related to drug resistance between HeLa/Dox vs K562/Dox cell lines

**Figure 4:**
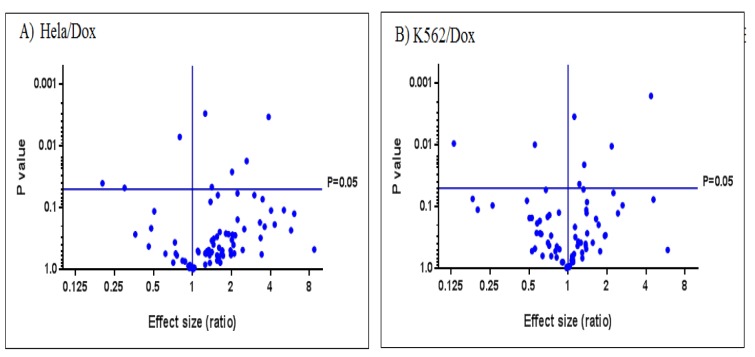
Volcano plots displaying differential expressed genes in Wnt signaling pathway between HeLa/Dox vs K562/Dox cell lines

Volcano plots of Figure 3(Fig. 3) and Figure 4(Fig. 4) display differentially expressed genes that are related to drug resistant and Wnt signaling pathway between doxorubicin resistant HeLa and K562 cell lines respectively.

**Figure 5:**
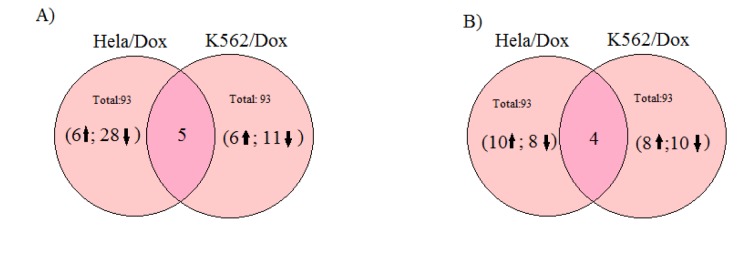
The intersection graph for commonly up- or down-regulated genes between doxorubicin resistant Hela and K562 cell lines A) Wnt signaling pathway, B) Drug resistance related genes

The intersection graph (Figure 5A(Fig. 5)) shows that there are five common downregulated genes in Wnt signaling pathway between doxorubicin resistant HeLa and K562 cell lines. On the other hand there are two commonly upregulated and two commonly downregulated genes that are related to drug resistance between doxorubicin resistant HeLa and K562 cell lines (Figure 5B(Fig. 5)).

## Discussion

Chemotherapy, as one of the most commonly used methods of treating cancer, the resistance to chemotherapeutic agents severely limits the success of the therapy. The data obtained from *in vitro *and* in vivo* studies reveal some important mechanisms for drug resistance. One of the most important mechanism has been identified as overexpression of ABC-transporter proteins found in the cell membrane which involved in the extracellular release of the drugs. These pump proteins prevent the accumulation of anticancer drugs into the cell, thereby preventing the expected toxic effect from occurring. Apart from this, different resistance mechanisms have also been identified. These include; increased metabolism of the drug, increased DNA repair, nonfunctional apoptosis mechanisms, and alterations of the expression or structure of the drug target molecules (Lage, 2008[18]). 

The role of Wnt signal transduction in the emergence of many cancer types and its relationship with chemotherapy resistance has been a subject of interest in recent years. There are three types of Wnt signaling pathways; Wnt/β-catenin (canonic), non-canonic and Wnt/calcium. Especially, the mechanisms between Wnt/β-catenin signal activation, blast transformation and drug resistance are being explored (Huelsken and Behrens, 2002[11]). 

In this study, attempts were made to identify changes in the expression levels of 186 genes which have important roles in drug resistance and Wnt signaling pathways in original and doxorubicin resistant HeLa and K562 cells. The selection of the model cell lines to two different types of cancer is to demonstrate possible similar and/or different effects of the same drug in different types of cancer.

Resistant variants of HeLa and K562 cell lines were obtained and resistance development were determined by XTT cytotoxicity analysis. According to the results, the doxorubicin resistance in the HeLa cell line was about 2-folds higher than that of the original HeLa line; and the doxorubicin-resistant K562 cell line developed about 32 times more resistance than the original K562 line. It is seen that the amount of resistance developed to the same drug is significantly different in different cancer cell lines. 

The expression of the ABCB1 gene encoding MDR1 protein in both cell lines was significantly increased to 160 and 103 folds, respectively, in doxorubicin resistant HeLa and K562 cell lines. In many studies, it has been shown that overexpression of MDR1 in cancer cells is responsible for resistance to chemotherapy drugs (Haber, 1992[10]). The increase in expression of MDR1 gene is responsible for doxorubicin resistance in human hepatocellular carcinoma cell lines (Park et al., 1994[32]); the increase in expression of MDR1 in human breast cancer has been shown to correlate with resistance to taxol and doxorubicin (Mechetner et al., 1998[23]). In another study conducted with blood samples from patients with acute myeloid leukemia, it was found that the increase in MDR1 expression was also responsible for resistance to daunorubicin, doxorubicin and etoposide (Nørgaard et al., 1998[27]). In addition to MDR1, MRP1 is another protein being responsible for resistance development in cancer treatment. Overexpression of MRP1 has been shown to be responsible for resistance to different anticancer agents used in the treatment of breast cancer patients (Zeng et al., 2001[39]; Goldhirsch et al., 2003[9]). In another study with doxorubicin-resistant prostate cancer cell lines 0.03 and PC 0.03, the mechanism responsible for resistance development was found to be MRP1 rather than MDR1 (Zalcberg et al., 2000[38]). According to our results, the decrease in the expression of the ABCC1 gene encoding the MRP1 in both cell lines suggests that the doxorubicin resistance can be due to an increase in the MDR1 gene rather than MRP1.

It is known that oncogen activation is related to the development of cancer formation and drug resistance. For this reason, many cancer drugs have been developed to target oncoproteins (Hunter, 1984[12]). It has been found that epidermal growth factor (EGF), platelet derived proliferation factor (PDGF) and their receptors are overexpressed (Ronellenfitsch et al., 2010[34]) in glioblastoma tumors; c-met and PDGFR are co-expressed in resistance mechanisms against EGFR inhibitors (Stommel et al., 2007[35]). Increased expression of EGFR which is induced by CGF (cancer upregulated gene 2) has been shown to induce doxorubicin resistance through the Stat1-HDAC4 signal in lung cancer cells (Kaowinn et al., 2017[15]). In addition to this information in the literature, in our previous studies with prednisone and vincristine resistant multiple myeloma cell lines have shown that different drugs cause different oncogene expressions on the same cancer cell line, and therefore, it is important to evaluate these data separately in drug resistance (Mutlu et al., 2012[26]). Similar to the literature, the data obtained from this study also support the literature, it is seen that the different oncogene expressions have changed in doxorubicin-resistant HeLa and K562 cell lines in spite of the resistance to the same drug. Overexpression of Fos and Myc genes were observed in HeLa/Dox and K562/Dox cell lines respectively. In addition, significant differences were determined in gene expressions that are involved in cell cycle, apoptosis and drug metabolism (CDKN1A, CCND1, BAX, FAS), growth factors and its receptors (FGF2, IGF1R, IGF2R, MET), different transcription factors (HIF1A, NFKB1, NFKB2, RANK), CYP2E1, CYP3A5) between doxorubicin-resistant HeLa and K562 cell lines. 

There are also significant differences between the doxorubicin-resistant HeLa and K562 cells in the expression of the genes involved in the Wnt signaling pathway. Among 93 genes included in this study; expression levels of 36 genes in HeLa/Dox and 17 genes in K562/Dox were observed to change with respect to drug-sensitive variants. Activation of Wnt signaling pathways begins with the binding of the Wnt protein to the FZD and LRP5/6 receptors in the target cell membrane. Some of the beta-catenin that accumulates in the cytoplasm enters the nucleus, causing transcription of target genes (Kikuchi et al., 2007[16]; Brembeck et al., 2006[3]). It has been found that due to the increase in the amount of beta-catenin, cyclin D1 and c-myc activation generates which leads to the resistance to lenidomide in multiple myeloma (Bjorklund et al., 2011[1]). In this study, when we look at doxorubicin-resistant K562 chronic myeloid leukemia cells, there was no significant change in expression of CTNNB1 gene encoding beta-catenin; as well as an increase of about 8-folds was observed in the gene encoding FZD1 from Wnt receptors. On the other hand, 15-folds decrease in the DAB2 gene and 3-folds decrease in the SOCS3 gene were determined which are encoding tumor suppressor proteins. Furthermore, it has been determined that there was a significant decrease in expression of the AXIN2 gene that is found in the beta-catenin degradation complex and in the expression of the CXXC4 gene that encodes a Wnt antagonist. In a study by Jamieson et al., there is an increase in beta-catenin expression in blast crisis-phase or imatinib-resistant CML cells (Jamieson et al., 2004[14]). The reason not to see a change in beta-catenin expression in doxorubicin-resistant K562 cells, can be the decrease in the expression of AXIN2 and Wnt antagonist molecule genes that cause beta-catenin degradation may lead to increased intracellular beta-catenin content and thus Wnt signal activation in K562/Dox cells.

It has been reported that FZD7 from Wnt receptors is associated with cell proliferation, metastasis and drug resistance in squamous cell carcinoma (Liu et al., 2017[19]). In HeLa/ Dox cells, it was generally found that there was a decrease in the expression of Wnt receptor genes. In addition, the GSK3B gene expression was found to be decreased as 2 folds and the expression of the ABCG2 gene from the ABC carrier protein family members increased 9-folds in HeLa/Dox cell line. This data supports the study which is related to colorectal cancer cells in the literature. According to Chen et al., due to the increase in miR-199a/b expression in cisplatin-resistant ALDHA1^+^ colorectal cancer stem cells, expression of GSK3B gene decrease whereas expression level of ABCG2 gene increase which is one of the lower target molecules of the Wnt signal pathway and is an important protein for drug resistance development (Chen et al., 2017[4]). In another study, it was also reported that ABCB1 and ABCG2 gene expression levels were significantly decreased and cells became sensitive to chemotherapy after silencing of beta-catenin gene in drug resistant colon cancer cell lines (Chikazawa et al., 2010[6]). Three folds decrease was shown in the expression level of caspase-9 gene that have a central role in apoptosis at HeLa/Dox cell line. This result suggests that HeLa cells become resistant to apoptosis by escaping from apoptosis. In addition, it is remarkable that the gene expression of the negative regulator molecules (DKK1, FBXW4, KREMEN1 and TLE1) in the Wnt signaling pathway in HeLa/Dox cells were significantly reduced.

In conclusion; this study provides important information on the level of gene expressions in terms of determining the relationship between doxorubicin resistance and changes in the Wnt signaling pathway in HeLa and K562 cell lines. However, in order to investigate these relations in more detail, functional analyzes at the protein level are required in the following studies.

## Funding

This work was supported by TÜBİTAK 3001 project (Project No: 214S634).

## Conflict of interest

The authors declare that they have no conflict of interest.

## Figures and Tables

**Table 1:**
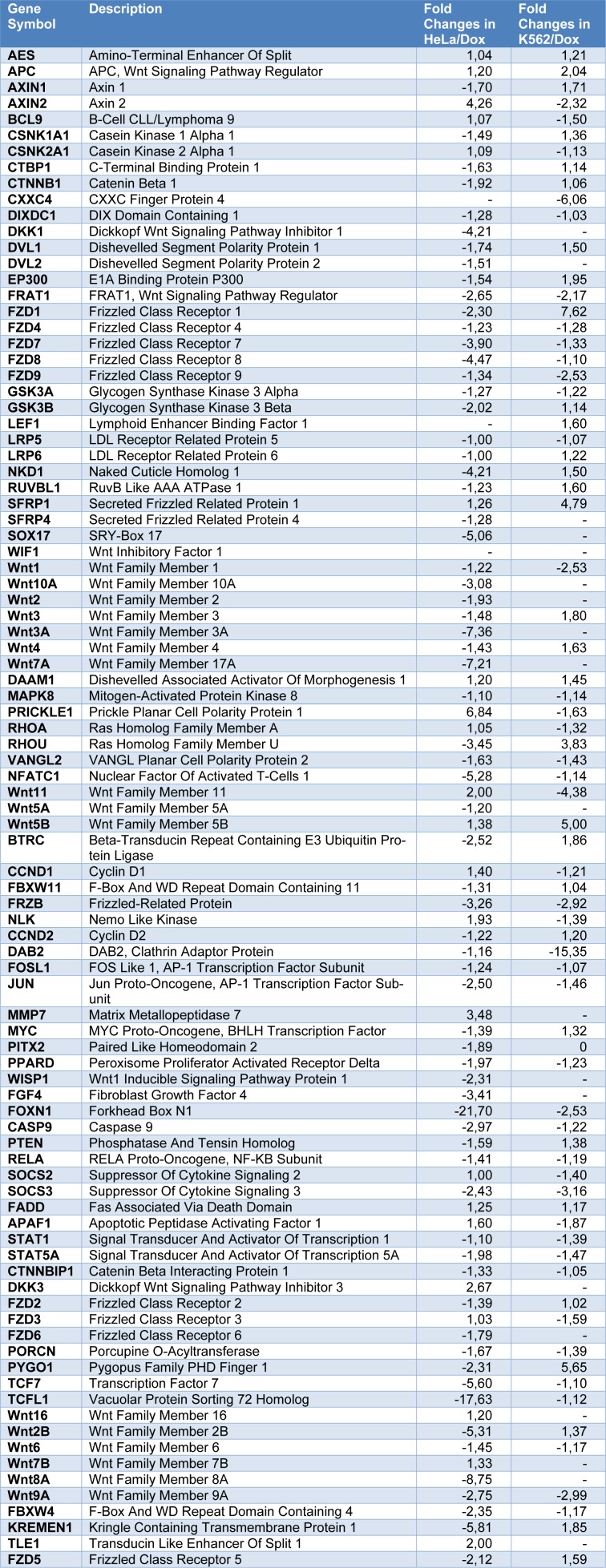
Changes in the gene expression levels of the Wnt signaling pathway

**Table 2:**
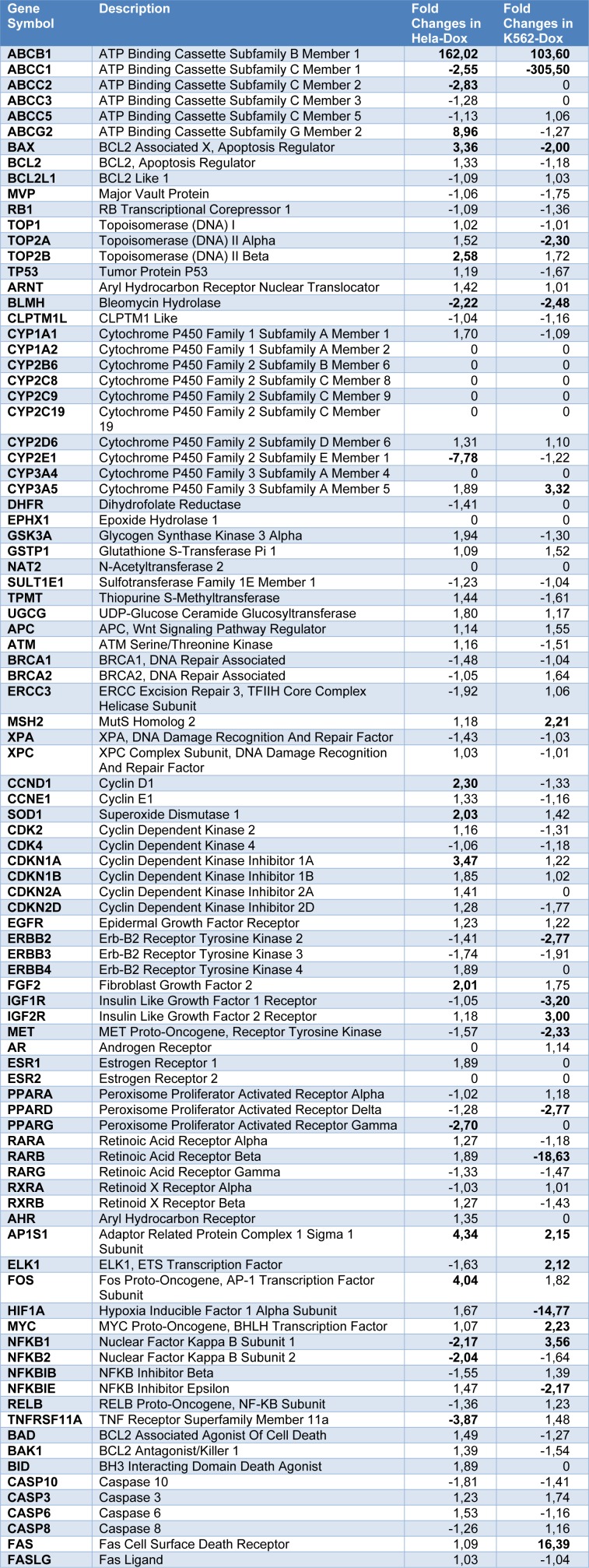
Changes in the expression levels of the genes that are related to drug resistance

## References

[1] Bjorklund CC, Ma W, Wang ZQ, Davis RE, Kuhn DJ, Kornblau SM (2011). Evidence of a role for activation of Wnt/β-Catenin signaling in the resistance of plasma cells to lenalidomide. J Biol Chem.

[2] Bourguignon LYW, Xia W, Wong G (2009). Hyaluronan-mediated CD44interaction with p300 and SIRT1 regulates beta-catenin signaling andNFkappaB-specific transcription activity leading to MDR1 and Bcl-xLgene expression and chemoresistance in breast tumor cells. J Biol Chem.

[3] Brembeck FH, Rosário M, Birchmeier W (2006). Balancing cell adhesion and Wnt signaling, the key role of beta-catenin. Curr Opin Genet Dev.

[4] Chen B, Zhang D, Kuai J, Cheng M, Fang X, Li G (2017). Upregulation of miR-119a/b contributes to cisplatin resistance via Wnt/β-catenin-ABCG2 signaling pathway in ALDHA1+ colorectal cancer stem cells. Tumor Biol.

[5] Chen W, Chen M, Barak LS (2010). Development of small molecules targeting the Wnt pathway for the treatmentof colon cancer: a high-throughput screening approach. Am J Physiol.

[6] Chikazawa N, Tanaka H, Tasaka T, Nakamura M, Tanaka M, Onishi H (2010). Inhibition of Wnt signaling pathway decreases chemotherapy-resistant side-population colon cancer cells. Anticancer Res.

[7] Flahaut M, Meier R, Coulon A, Nardou KA, Niggli FK, Martinet D (2009). The Wnt receptor FZD1 mediateschemoresistance in neuroblastoma through activation of the Wnt/beta-catenin pathway. Oncogene.

[8] Ge X, Wang X (2010). Role of Wnt canonical pathwayin hematological malignancies. J Hematol Oncol.

[9] Goldhirsch A, Wood WC, Gelber RD, Coates AS, Thürlimann B, Senn HJ (2003). Meeting highlights: Updated international expert consensus on the primary therapy of early breast cancer. J Clin Oncol.

[10] Haber DA (1992). Multidrug resistance (MDR1) in leukemia: is it time to test?. Blood.

[11] Huelsken J, Behrens J (2002). The Wnt signalling pathway. J Cell Sci.

[12] Hunter T (1984). The proteins of oncogenes. Sci Am.

[13] Ilyas M (2005). Wnt signalling and the mechanistic basis of tumour development. J Pathol.

[14] Jamieson CH, Ailles LE, Dylla SJ, Muijtjens M, Jones C, Zehnder JL (2004). Granulocyte-macrophage progenitors as candidate leukemic stem cells in blast-crisis. N Engl J Med.

[15] Kaowinn S, Jun SW, Kim CS, Shin DM, Hwang YH, Kim K (2017). Increased EGFR expression induced by a novel oncogene, CUG2, confers resistance to doxorubicin through Stat1-HDAC4 signaling. Cell Oncol (Dordr).

[16] Kikuchi A, Yamamoto H, Kishida S (2007). Multiplicity of the interactions of Wnt proteins and their receptors. Cell Signal.

[17] Krishan A, Fitz CM, Andritsch I (1997). Drug retention, efflux, and resistance in tumor cells. Cytometry.

[18] Lage H (2008). An overview of cancer multidurg resistance: a still unsolved problem. Cell Mol Life Sci.

[19] Liu X, Yan Y, Ma W, Wu S (2017). Knockdown of frizzled-7 inhibits cell growth and metastasis and promotes chemosensitivity of esophageal squamous cell carcinoma cells by inhibiting Wnt signaling. Biochem Biophys Res Commun.

[20] Lu W, Tinsley HN, Keeton A, Qu Z, Piazza GA, Li Y (2009). Suppression of Wnt/β-catenin signaling inhibits prostatecancer cell proliferation. Eur J Pharmacol.

[21] Lustig B, Behrens JJ (2003). The Wnt signaling pathway and its role in tumor development. J Cancer Res Clin Oncol.

[22] Marie J-P (2001). Drug resistance in hematologic malignancies. Curr Opin Oncol.

[23] Mechetner E, Kyshtoobayeva A, Zonis S, Kim H, Stroup R, Garcia R (1998). Levels of multidrug resistance (MDR1) P-glycoprotein expression by human breast cancer correlate with in vitro resistance to taxol and doxorubicin. Clin Cancer Res.

[24] Mikels AJ, Nusse R (2006). Wnts as ligands: processing, secretion and reception. Oncogene.

[25] Miller JR (2002). The Wnts. Genome Biol.

[26] Mutlu P, Ural AU, Gündüz U (2012). Differential oncogene-related gene expressions in myeloma cells resistant to prednisone and vincristine. Biomed Pharmacother.

[27] Nørgaard JM, Bukh A, Langkjer ST, Clausen N, Palshof T, Hokland P (1998). MDR1 gene expression and drug resistance of AML cells. Br J Haematol.

[28] Nusse R (2005). Wnt signaling in disease and in development. Cell Res.

[29] Nusse R, Van Ooyen A, Cox D, Fung YK, Varmus H (1984). Mode of proviral activation of a putative mammary oncogene (int-1) on mouse chromosome 15. Nature.

[30] Nusse R, Varmus HE (1982). Many tumors induced by the mouse mammary tumor virus contain a provirus integrated in the same region of the host genome. Cell.

[31] Nusse R, Varmus HE (1992). Wnt genes. Cell.

[32] Park JG, Lee SK, Hong IG, Kim HS, Lim KH, Choe KJ (1994). MDR1 gene expression: its effect on drug resistance to doxorubicin in human hepatocellular carcinoma cell lines. J Natl Cancer Inst.

[33] Prosperi JR, Goss KH (2010). A Wnt-ow of opportunity: targeting the Wnt/β-catenin pathway in breast cancer. Curr Drug Targets.

[34] Ronellenfitsch MW, Steinbach JP, Wick W (2010). Epidermal growth factor receptor and mammalian target of rapamycin as therapeutic targets in malignant glioma: current clinical status and perspectives. Target Oncol.

[35] Stommel JM, Kimmelman AC, Ying H, Nabioullin R, Ponugoti AH, Wiedemeyer R (2007). Coactivation of receptor tyrosine kinases affects the response of tumor cells to targeted therapies. Science.

[36] Ueda K, Cardarelli C, Gottesman MM, Pastan I (1987). Expression of a full-length cDNA for the human “MDR1” gene confers resistance to colchicine, doxorubicin, and vinblastine. Proc Natl Acad Sci U S A.

[37] Willert K, Nusse R (1998). Beta-catenin: a key mediator of Wnt signaling. Curr Opin Genet Dev.

[38] Zalcberg J, Hu XF, Slater A, Parisot J, El-Osta S, Kantharidis P (2000). MRP1 not MDR1 gene expression is the predominant mechanism of acquired multidrug resistance in two prostate carcinoma cell lines. Prostate Cancer Prostatic Dis.

[39] Zeng H, Chen ZS, Belinsky MG, Rea PA, Kruh GD (2001). Transport of methotrexate (MTX) and folates by multidrug resistance protein (MRP) 3 and MRP1: Effect of polyglutamylation on MTX transport. Cancer Res.

[40] Zhang H, Zhang X, Wu X, Li W, Su P, Cheng H (2012). Interference of Frizzled 1 (FZD1) reverses multidrug resistance in breast cancer cells through the Wnt/ß-catenin pathway. Cancer Lett.

